# Downregulation in GATA4 and Downstream Structural and Contractile Genes in the db/db Mouse Heart

**DOI:** 10.5402/2012/736860

**Published:** 2012-03-13

**Authors:** Tom L. Broderick, Marek Jankowski, Donghao Wang, Bogdan A. Danalache, Cassandra R. Parrott, Jolanta Gutkowska

**Affiliations:** ^1^Laboratory of Diabetes and Exercise Metabolism, Department of Physiology, Midwestern University, 19555 North 59th Avenue, Glendale, AZ, 85308, USA; ^2^Laboratory of Cardiovascular Biochemistry, Centre Hospitalier de L'Université de Montréal-Hôtel-Dieu, 3850 St-Urbaiw Street, Montreal, QC, Canada H2W 1T8

## Abstract

Reduced expression of GATA4, a transcriptional factor for structural and cardioprotective genes, has been proposed as a factor contributing to the development of cardiomyopathy. We investigated whether the reduction of cardiac GATA4 expression reported in diabetes alters the expression of downstream genes, namely, atrial natriuretic peptide (ANP), B-type natriuretic, peptide (BNP), and **α**- and **β**-myosin heavy chain (MHC). db/db mice, a model of type 2 diabetes, with lean littermates serving as controls, were studied. db/db mice exhibited obesity, hyperglycemia, and reduced protein expression of cardiac GLUT4 and IRAP (insulin-regulated aminopeptidase), the structural protein cosecreted with GLUT4. Hearts from db/db mice had reduced protein expression of GATA4 (*~*35%) with accompanying reductions in mRNA expression of ANP (*~*40%), BNP (*~*85%), and **α**-MHC mRNA (*~*50%) whereas expression of **β**-MHC mRNA was increased by *~*60%. Low GATA4 was not explained by an increased ligase or atrogin1 expression. CHIP protein content was modestly downregulated (27%) in db/db mice whereas mRNA and protein expression of the CHIP cochaperone HSP70 was significantly decreased in db/db hearts. Our results indicate that low GATA4 in db/db mouse heart is accompanied by reduced expression of GATA4-regulated cardioprotective and structural genes, which may explain the development of cardiomyopathy in diabetes.

## 1. Introduction

Diabetes is a major risk factor in the development of cardiovascular disease [1]. Impairment of left ventricular function is frequent in patients with type 2 diabetes even in the absence of ischemic, hypertensive, and valvular heart disease [2, 3]. Type 2 diabetes is associated with increased adverse outcomes following ischemic events, reflected by a heightened mortality rate [4]. Although disturbances in energy metabolism and vascular endothelial function play a role in the development of left ventricular dysfunction, altered transcription of genes encoding for contractile and structural proteins also contribute to the cardiovascular risk of diabetic patients [5–8].

GATA4 is a zinc finger-containing transcription factor that belongs to the GATA superfamily [9]. GATA4 is highly expressed in cardiomyocytes where it regulates the transcription of *α*- and *β*-myosin heavy chain (MHC) composition, atrial natriuretic (ANP), and B-type natriuretic (BNP) peptides, which are important in cardiac function, blood pressure regulation, and cardioprotection [9, 10]. Accordingly, GATA4 is essential for important adaptive responses such as cardiomyocyte survival, hypertrophy in response to pressure overload and exercise, and protection against ischemic insult [11–14]. In failing rat hearts, GATA4 levels are markedly reduced and recent evidence indicates that cardiac GATA4 degradation is increased in diabetes [15, 16].

In hearts from diabetic db/db mice, low levels of GATA4 are thought to occur as a result of increased E-3 ubiquitin ligase carboxyl terminus of Hsp70-interacting protein (CHIP) activity [16]. Alternatively, a recent study demonstrated the importance of CHIP and its cochaperone heat shock protein 70 (HSP70), in the critical rescue of the myocardium from acute ischemia. In mouse hearts from CHIP-deleted mice, HSP levels are correspondingly low, and an increased susceptibility to ischemic injury is observed as left ventricular function is compromised [17]. Based on this evidence, low GATA4 levels in the db/db heart would suggest that the documented cardiomyopathy in this model of diabetes may be accompanied by a downregulation of GATA4-regulated structural and cardioprotective genes.

Stimulation of the oxytocin receptor is a key component of a cardioprotective system associated with ANP, BNP, and nitric oxide synthesis, and stimulation of glucose uptake [18–21]. Considering the role of GATA4 in the regulation of these natriuretic peptides and contractile proteins, a reduction in cardiac GATA4 would suggest that the synthesis of these genes is altered by the diabetic state. Thus, in the present study, we hypothesize that hearts from db/db mice exhibit specific disturbances in the expression of structural and cardioprotective genes resulting from low GATA4 levels. To test this hypothesis, we used the db/db mouse model of diabetes because of its close representation of human type 2 diabetes and also because the GATA4-regulated downstream genes in this model of diabetes have not been determined. The onset of diabetes in the db/db mouse is gradual and is characterized by obesity, hyperglycemia, hyperinsulinemia, and insulin resistance and by 12–16 weeks of age, hearts demonstrate increased susceptibility to ischemic injury, cardiomyopathy from increased fibrosis, collagen accumulation and increased apoptosis, and left ventricular dysfunction [22–24].

## 2. Materials and Methods

### 2.1. Mouse Model of Diabetes

The Midwestern University Research and Animal Care Committee approved this study. All animals used in this study were cared, in accordance to the recommendations in The Guide for the Care and Use of Laboratory Animals, National Institute of Health, Publ. No. 85-23, 1986. Diabetic mice (C57BL/KsJ-lept^db^-lept^db^) were obtained from Jackson Laboratories (Bar Harbor, ME) and studied at the age of 14 weeks. The db/db mouse displays many of the metabolic perturbations associated with type 2 diabetes as a result of two mutant copies of the leptin receptor gene. The lean littermates, which possess one mutant and one normal copy of the leptin (db/^+^), were used as controls. Mice were provided with food and water were provided *ad libitum* and maintained in a room with alternating twelve-hour light/dark cycle and kept at 22°C.

### 2.2. Measurement of Blood Pressure

 Systolic and diastolic pressures were measured from restrained mice with a pneumatic tail-cuff device (NIBP-8, Columbus instruments, Columbus, OH). From these measurements, mean arterial pressure (MAP) and heart rate were determined. Blood pressure readings were obtained two days before the mice were sacrificed.

### 2.3. Blood and Tissue Sampling

Overnight-fasted mice were sacrificed in the morning between 8 and 11 AM. Blood was obtained from the mandibular vein, and then mice were immediately sacrificed by cervical dislocation. Blood was immediately centrifuged (3,000 rpm at 4°C, for 5 min) and plasma separated from the erythrocytes for the assay of glucose (Wako Chemical, VA). The packed erythrocytes were used for the determination of glycosylated haemoglobin (Helena Laboratories, TX).

Hearts were rapidly removed and frozen with clamps precooled to the temperature of liquid N_2_ for analysis of GATA4, ANP, BNP, *α*- and *β*-MHC, Nab1, heat shock protein70 (HSP70), glucose transporter protein (GLUT4), insulin-regulated aminopeptidase (IRAP), and the following E3 ubiquitin ligases: carboxyl terminus of Hsp 70-interacting protein (CHIP), muscle ring finger protein-1 (MuRF1), and atrogin1 genes. Frozen tissue was first ground to powder under liquid nitrogen and then thoroughly homogenized using a Teflon pestle in a glass homogenization tube cooled in ice.

### 2.4. Cell Morphology and Imaging

Primary cardiomyocytes cultures were prepared from ventricles of 2-day-old Sprague Dawley rats using the Neonatal Cardiomyocyte Isolation System (Cat. No. LK003300; Worthington, Lakewood, NJ) as reported previously [20]. The cells were incubated 24 h in 0.01% poly-LlLysine (Cat. no. P4832-covered Lab-Tek plates, Sigma-Aldrich; Cat. no. 177437, Nunc International, Rochester, NY) for microscopic analysis. Tissuefix solution (Laboratory Gilles Chaput Inc., Montreal, Quebec) was used for cell fixation. Primary anti- GATA-4 (c-20) goat antibody (Cat. no. sc-1237, Santa Cruz Biotechnology, Santa Cruz, CA) was used at 1 : 250 dilution. Secondary donkey anti-goat IgG secondary antibody conjugated to red fluorophore Alexa Fluor 594 was obtained from Invitrogen (Cat. no. A11058, Life Technologies, Carlsbad, CA). GATA4 was costained with troponin C using mouse monoclonal antibody against cardiac troponin (1 : 100, Cat. no. ab7217-7, Abcam, Cambridge, MA). Green secondary donkey anti-mouse IgG antibody Alexa Fluor 488 was purchased from Invitrogen (Cat. no. A21202, Life Technologies, Carlsbad, CA). Mounting medium DAPI (4′,6-diamidino-2-phenylindole) with P7481 antifade reagent (Invitrogen, Cat. No. P7481, Life Technologies, Carlsbad, CA) was used for the identification of the nuclei. Cell morphology was examined under a Model IX51 inverted microscope (Olympus, Tokyo, Japan). Micrographs were taken with a Q Imaging QICAM-IR Fast 1394 Digital CCD camera. To measure cardiomyocyte size, the surface of 50 cells was recorded manually using at least 7 photographs and then calculated in *μ*m^2^. Micrographs and cardiomyocyte cell size were analyzed using Image J software (National Institutes of Health, Bethesda, MD).

### 2.5. Real-Time PCR

 Total RNA was extracted from freeze-clamped hearts with Trizol reagent (Invitrogen Life Technologies, Burlington, ON) according to the manufacturer's protocol. To remove genomic DNA, RNA samples were incubated with 2 U deoxyribonuclease I (DNase I; Invitrogen Life Technologies, Burlington, ON)/*μ*g RNA for 30 min at 37°C. PCR was carried out in the iCycler IQ real-time PCR detection system (Bio-Rad Laboratories, Hercules, CA) using SYBR green chemistry. The samples were analysed in duplicate or triplicate. For amplification, 2 *μ*L of diluted cDNA were added to a 20 *μ*L reaction mixture containing 1X iQ SYBR Green Supermix (Bio-Rad Laboratories, Hercules, CA) and 200 nM forward and reverse primers. The thermal cycling program was 95°C for 2 min, followed by 40 cycles of 95°C for 25 s, 60°C for 25 s, and 72°C for 25 s. The primers were purchased from Invitrogen Life Technologies (Burlington, ON). Primer sets served to generate amplicons (Table 1). Optical data were recorded during the annealing step of each cycle. After PCR, the reaction products were melted for 1 min at 95°C, the temperature was lowered to 55°C, and then gradually increased to 95°C in 1.0°C increments, 10 s per increment. Optical data were collected over the duration of the temperature increments, with a dramatic drop in fluorescence occurring. This was done to ensure that only 1 PCR product was amplified per reaction.

The relative expression of the RT-PCR products was determined by the ΔΔCt method. This method calculates relative expression using the equation: fold induction = 2^-[ΔΔCt]^, where Ct = the threshold cycle, that is, the cycle number at which the sample's relative fluorescence rises above background fluorescence and ΔΔCt = [Ct gene of interest (unknown sample)−Ct GAPDH (unknown sample)]−[Ct gene of interest (calibrator sample)−Ct GAPDH (calibrator sample)]. One of the control samples was chosen as the calibrator sample and tested in each PCR. Each sample was run in duplicate, and mean Ct was taken in the ΔΔCt equation. GAPDH was chosen for normalization because this gene showed consistent expression relative to other housekeeping genes among the treatment groups in our array experiments.

### 2.6. Western Blot Analysis

Heart samples (~100 mg) were prepared by homogenisation in modified RIPA buffer (1 × PBS, 1% Igepal CA-630, 0.5% sodium deoxycholate, 0.1% SDS, 10 mg/mL PMSF, aprotinin, 100 mM sodium orthovanadate and 4% protease inhibitor). After 2 hours in constant agitation at 4°C, the samples were centrifuged at 10,000 g for 20 min at 4°C. The supernatants were collected and the protein concentration was determined by a modified Bradford assay. Thirty micrograms of total protein were applied to each well of 10% SDS polyacrylamide gel and electrophoresed for 2 h at 130 V (MHC: 20 h at 140 v) along with a set of molecular weight markers (RPN800, Amersham Biosciences, Baie dUrfe, PQ). The resolved protein bands were then transferred onto PVDF membranes (Hybond-C; Amersham Pharmacia Biotech Inc., Piscataway, NJ) at 20 V for 60 min at room temperature using a transfer buffer (25 mmol/L Tris base, 192 mmol/L glycine, and 20% methanol). The blots were blocked overnight at 4°C with blocking buffer consisting of 5% nonfat milk in 10 mmol/L Tris pH 7.5, 100 mmol/L NaCl, 0.1% Tween 20 (Amersham Pharmacia Biotech Inc, Piscataway, NJ). The membranes were then probed with specific primary antibodies: GATA4 (1 : 500, sc-25310, Santa Cruz Biotechnology, Santa Cruz, CA), Nab1 (1 : 1000, sc-12147, Santa Cruz Biotechnology), HSP70 (1 : 10,000, sc-32239, Santa Cruz Biotechnology), CHIP (1 : 2000, sc-133083, Santa Cruz Biotechnology), GLUT4 (1 : 10000, 4670-1725, AbD), IRAP (1 : 10000, kindly given by Dr. Pilch, Boston University School of Medicine, MA), MHC (1 : 5000, NB 300-284, Novus Biologicals, Littleton, CO) overnight at 4°C. As an internal control, blots were reprobed with an anti-*β*-GAPDH antibody (1 : 20000; G9545-200UL, Sigma-Aldrich). Blots were then washed using TBS washing buffer (10 mmol/L Tris pH 7.5, 100 mmol/L NaCl, 0.1% Tween 20) and incubated with horseradish peroxidase-conjugated immunoglobulin G (IgG) (anti-mouse for GATA4, GLUT4, HSP70, and MHC, 1 : 10000; anti-goat for Nab1, 1 : 10000; anti-rabbit for IRAP and GAPDH, 1 : 10000) during 1 h at room temperature. The blots finally were detected by chemiluminescence detection system (RPN2132, Amersham Biosciences, Baie dUrfe, PQ) and visualized by exposure to Kodak X-Omat film. Densitometric measurement of the bands was performed using Photoshop 7 software.

### 2.7. Statistical Analysis

 The statistical analysis was performed using the statistical software package Prism 3.0. The unpaired *t*-test was used to determine differences between group means. All values are expressed as mean ± SEM with significance defined as *P* < 0.05.

## 3. Results

### 3.1. Physical Characteristics, Blood Pressure, GLUT4, and IRAP Expression in db/db Mice

**Figure 1:**
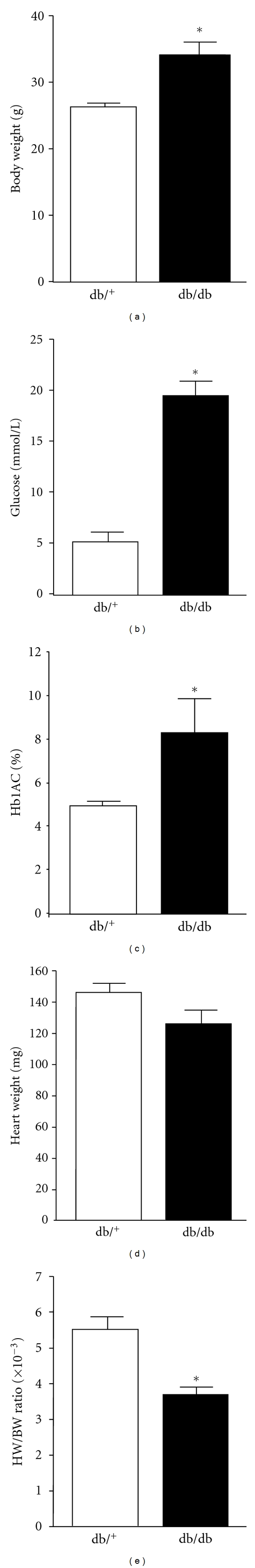
Body weight (a), plasma glucose (b), glycated haemoglobin (c), heart weight (d), and the heart-weight-to-body-weight ratio (e) in control and db/db mice. Values are expressed as mean ± SEM for 10–12 mice in each group. db/^+^, control mice; db/db, diabetic mice. *P < 0.05.

**Figure 2:**
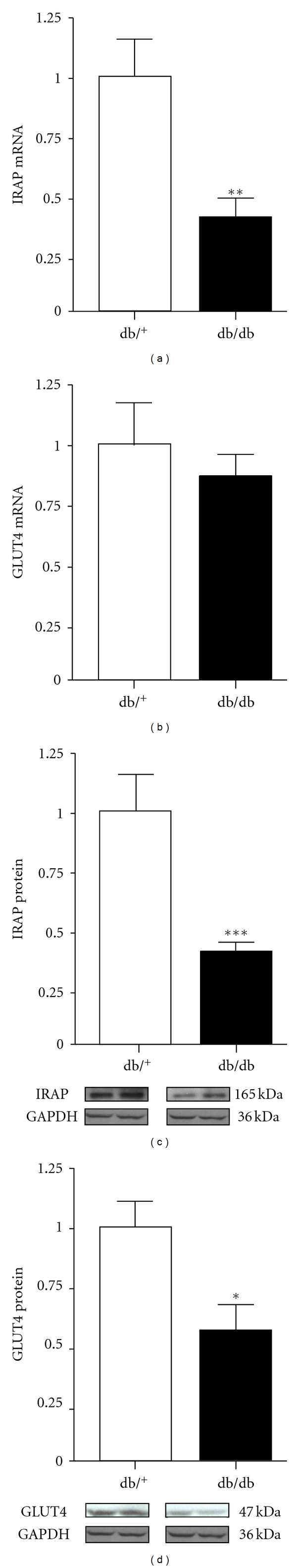
Cardiac GLUT4 and IRAP mRNA and protein expression in control and db/db mice. Values are expressed as mean ± SEM obtained from 2 separate experiments each performed with 5 hearts. db/^+^, control mice; db/db, diabetic mice. *P < 0.05, **P < 0.01, ***P < 0.001.

The physical characteristics of db/db mice are illustrated in Figure 1. Body weight, plasma glucose, and glycated (Hb_1AC_) haemoglobin levels were all significantly higher in db/db mice compared with control mice, confirming the typical phenotype of this model of diabetes. Although heart weight in db/db mice was similar to compared with control mice, the heart-to-body-weight ratio was significantly lower. Also consistent with hearts from db/db mice is a significant decrease (40%, *P* < 0.05) in protein expression of cardiac GLUT4 compared with control mice, as shown in Figure 2. This reduction in GLUT4 was associated with a concomitant decrease in mRNA (60%, *P* < 0.01) and protein expression (75%, *P* < 0.001) of IRAP which codistributes with GLUT4.

Blood pressure obtained in control (*n* = 6) and db/db mice (*n* = 5) demonstrated that systolic pressure was significantly higher in db/db mice compared with control mice (115 ± 2 versus 101 ± 5 mmHg, *P* < 0.05). However, there were no differences in diastolic pressure (82 ± 4 versus 76 ± 5 mmHg), MAP (92 ± 5 versus 84 ± 5 mmHg), and heart rate (475 ± 15 versus  499 ± 34  beats/min) between db/db mice and control mice, respectively.

### 3.2. GATA4 and Expression of Downstream Genes in db/db Hearts

**Figure 3:**
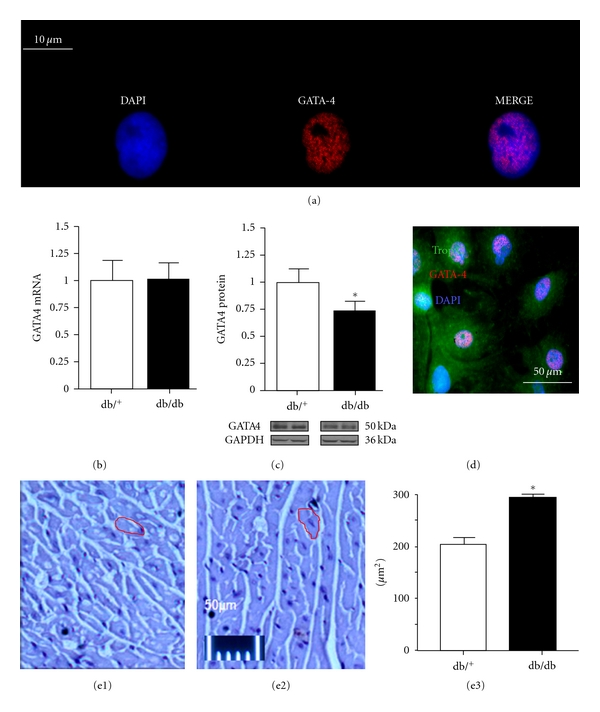
GATA4 is localized in the cell nuclei of cardiomyocytes of 2-day-old rats (a). GATA4 mRNA is expression is similar to that seen in control hearts (b). However, as illustrated in (c), protein expression of GATA4 is downregulated in db/db hearts (c). GATA4 was present in nuclei of the cells expressing cardiomyocyte marker, troponin C (d). Hearts from db/db mice showed a tendency toward a reduced weight (d), but cardiomyocyte surface was increased in db/db hearts (Figure 3e2) compared with control hearts (Figure 3e1). Figure (e3) shows that cardiomyocyte surface area, expressed as *μ*m^2^, was significantly increased in db/db hearts compared with control hearts. Values are expressed as mean ± SEM obtained from 2 separate experiments each performed with 5 hearts. db/^+^, control mice; db/db, diabetic mice. *P < 0.05.

 As illustrated in Figure 3(a), GATA4 is localized in the cell nuclei. Gene expression of GATA4 was not altered in db/db hearts (Figure 3(b)). However, a significant reduction (27%, *P* < 0.05) in GATA4 protein expression was observed in db/db hearts compared with control hearts (Figure 3(c)). Figure 3(d) shows that GATA4 was present in nuclei of cells expressing the cardiomyocyte marker, troponin C. Hearts from db/db mice showed a tendency towards a lower weight (Figure 1(d)), but cardiomyocyte enlargement was observed in these hearts (Figure 3(e2), 3(e3)) compared with control hearts (Figure 3(e1)).

**Figure 4:**
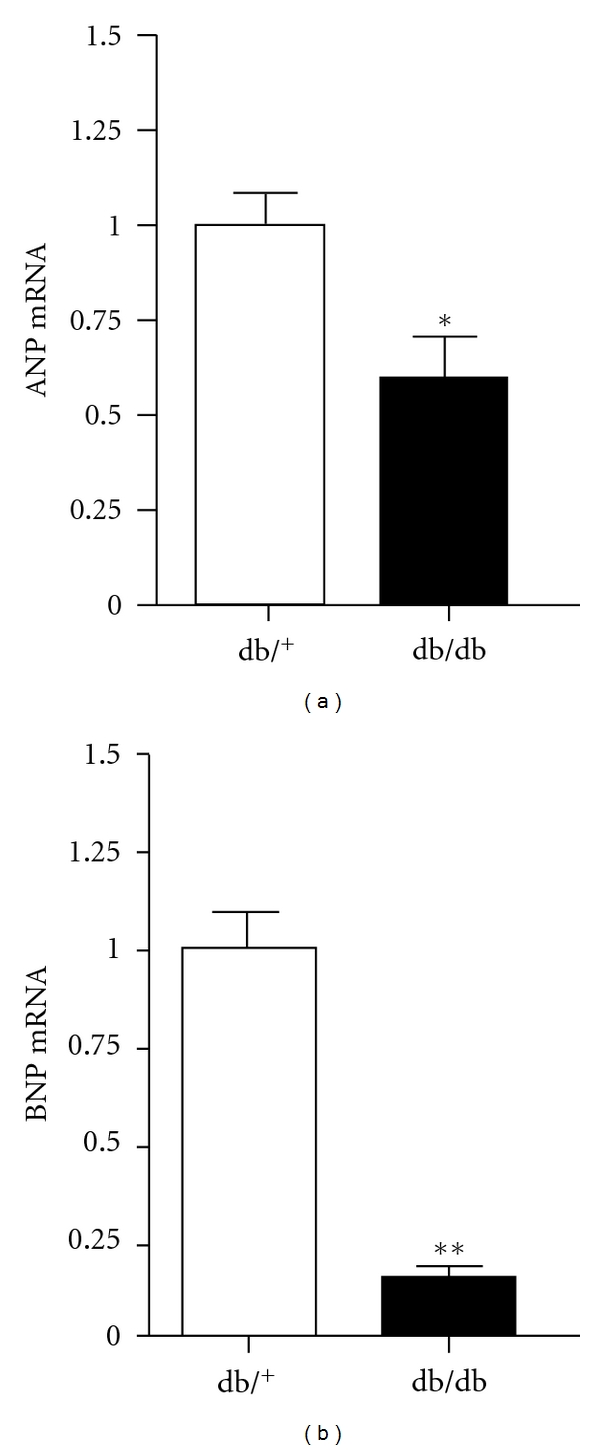
Cardiac ANP and BNP mRNA expression in control and db/db mice. Values are expressed as mean ± SEM obtained from 2 separate experiments each performed with 5 hearts. db/^+^, control mice; db/db, diabetic mice. *P < 0.05, **P < 0.01.

**Figure 5:**
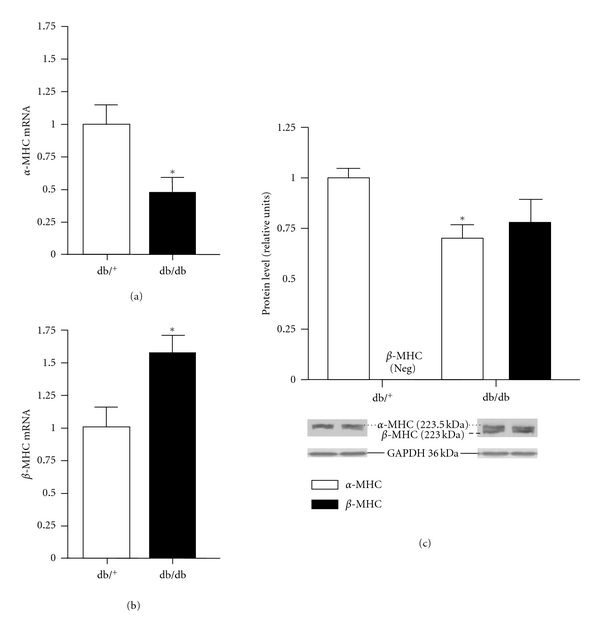
Cardiac *α*-MHC mRNA expression (a) *β*-MHC mRNA expression (b), and protein expression (c) in control and db/db mice. Protein expression of *β*-MHC was not detected in control hearts, whereas in db/db hearts, *β*-MHC expression was elevated. Values are expressed as mean ± SEM obtained from 2 separate experiments each performed with 5 hearts. db/^+^, control mice; db/db, diabetic mice. *P < 0.05.

The consequences of reduced cardiac GATA4 expression on downstream genes of interest are shown in Figures 4 and 5.  Figure 4 shows that the reduction in GATA4 in db/db hearts was associated with downregulation in the mRNA expression of ANP by 40% (*P* < 0.05) and BNP by 85% (*P* < 0.01) compared with control hearts. As illustrated in Figure 5, mRNA expression of *α*-MHC in db/db hearts was reduced by nearly 50% (*P* < 0.05) whereas *β*-MHC expression was increased by ~60% (*P* < 0.05). Compared with control hearts, a significant reduction (*P* < 0.05) in protein expression of *α*-MHC was observed in db/db hearts. Protein expression of *β*-MHC in control hearts was not detected, which is consistent with the observation that the dominant isoform consists of mainly the *α*-MHC in nondiseased hearts [25]. However, expression of *β*-MHC was markedly increased in db/db hearts.

### 3.3. Ubiquitin Ligase CHIP, MuRF1, Atrogin1, and Nab-1 Expression in db/db Hearts

**Figure 6:**
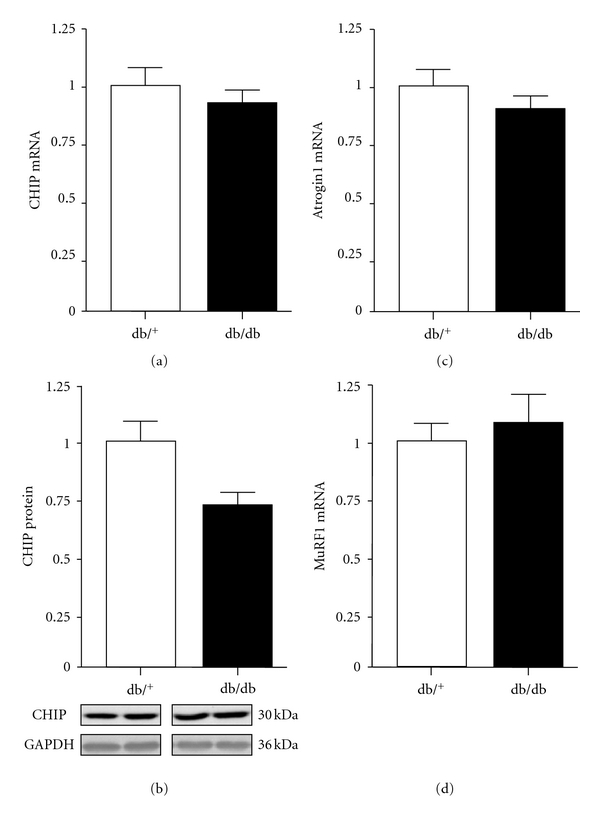
Cardiac CHIP mRNA and protein expression (a, b), atrogin1 mRNA expression (c), and MuRF1 mRNA expression (d) in control and db/db mice. Values are expressed as mean ± SEM obtained from 2 separate experiments each performed with 5 hearts. db/^+^, control mice; db/db, diabetic mice.

**Figure 7:**
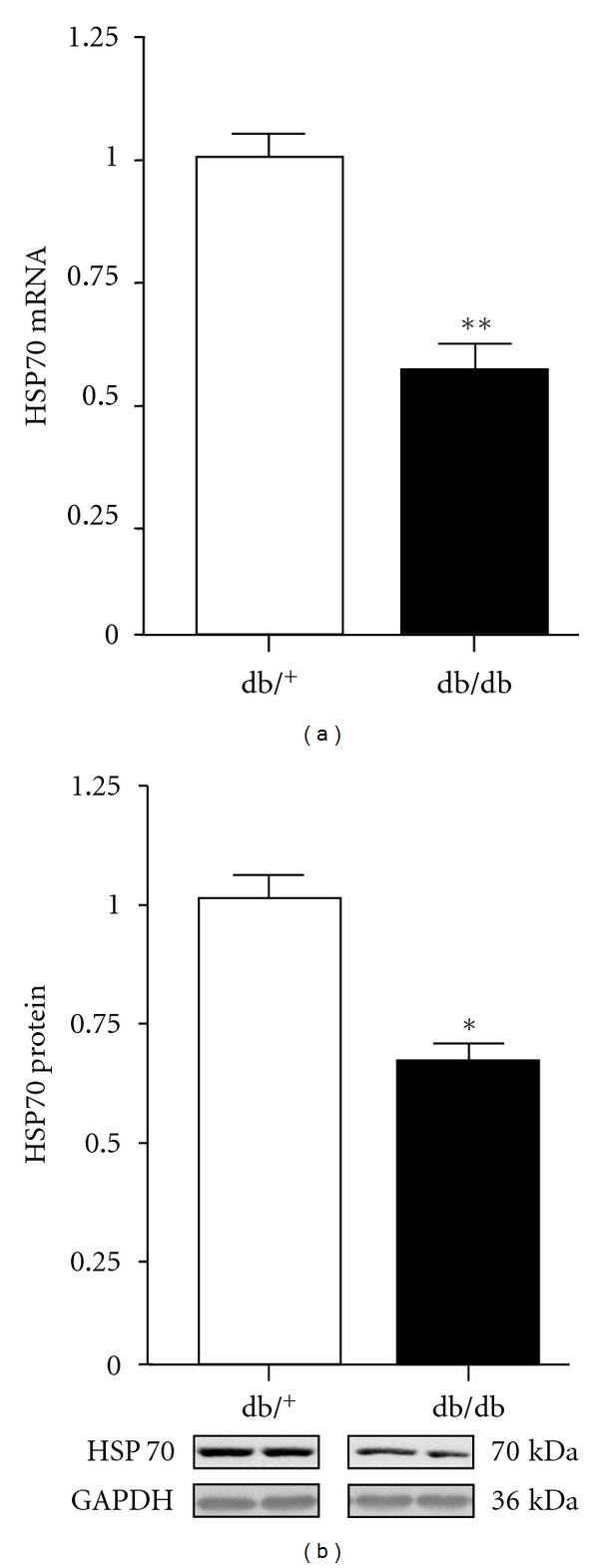
Cardiac HSP70 mRNA and protein expression in control and db/db mice. Values are expressed as mean ± SEM obtained from 2 separate experiments each performed with 5 hearts. db/^+^, control mice; db/db, diabetic mice. *P < 0.05, **P < 0.01.

To investigate whether the decrease in GATA4 protein expression in hearts of db/db mice was the result of increased proteosome activity, expression of ubiquitin ligases of CHIP, MuRF1, and atrogin1 were measured. As illustrated in Figure 6, no significant differences between db/db and control hearts were observed in the expression of these ligases, although CHIP protein expression was decreased by 26% in db/db hearts. However, we measured the molecular chaperone HSP70 because of its association with CHIP, and as shown in Figure 7, both HSP70 mRNA and protein expression were decreased by 45% and 35%, respectively, in db/db hearts compared with control hearts (Figure 7).

**Figure 8:**
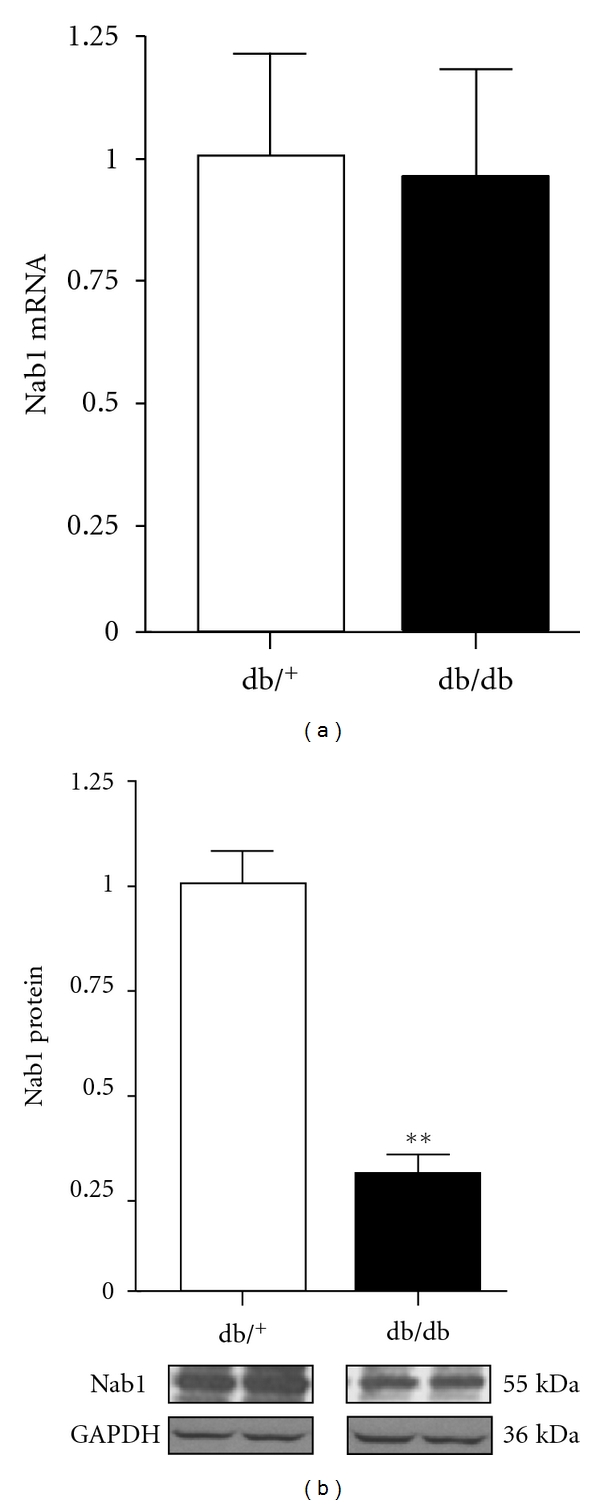
Cardiac Nab-1 mRNA and protein expression in control and db/db mice. Values are expressed as mean ± SEM obtained from 2 separate experiments each performed with 5 hearts. db/^+^, control mice; db/db, diabetic mice. **P < 0.01.

The cardiac hypertrophy marker Nab-1 was measured in db/db hearts. Nab1 (NGF1A-binding protein) is a member of a family of corepressors for early growth response (Egr) transcription factors that interacts with the inhibitory R1 repression domain of Egr1, thus acting as an endogenous regulator of pathological cardiac growth. As shown in Figure 8, mRNA levels were not altered in db/db hearts. However, Nab-1 protein levels were reduced by 65% (*P* < 0.01) in db/db hearts compared with control hearts.

## 4. Discussion

The db/db mouse is a widely accepted model for studying the consequences of type 2 diabetes on metabolic and cardiovascular function because this model shares several features with the human condition. The mice used in this study were obese, chronically hyperglycemic, and were studied at an age when hearts demonstrate alterations in structure and deficiencies in ventricular performance [22, 24]. A recent study demonstrated that acute hyperglycemia and the diabetic conditions induce degradation of cardiac GATA4 [16], an abundant transcription factor in heart that functions mainly as regulator of ANP, BNP, and *α*- and *β*-MHC expression [9, 10]. However, the consequences of reduced cardiac GATA4 levels on expression of these structural genes and peptides in the db/db model of type 2 diabetes have not been determined. Our main results demonstrate that the low GATA4 protein levels in db/db hearts validate its regulatory role on the mRNA expression of ANP, BNP, and *α*-MHC, indicating that these changes in target gene expression are consistent with the transcriptional role of GATA4. One exception, however, is that *β*-MHC expression was increased in db/db hearts. Further, we show that decreased GATA4 levels in db/db hearts are not caused by increased E3-ubiquitin proteosome function using the ligases MuRF1, atrogin1, and CHIP as markers. On the other hand, a significant decrease in CHIP molecular cochaperone HSP70 was observed in db/db hearts.

It is unclear why GATA4 protein levels are low in the db/db heart and what the mechanisms involved in this response are. Hyperglycemia per se has been previously proposed as a mechanism depleting cardiac GATA4 [16]. In conditions acutely mimicking the diabetic state with hyperglycemia or with chronic diabetes, degradation of cardiac GATA4 levels is accelerated. This degradation is thought to be mediated by ubiquitination through increased expression of the ubiquitin proteasome system [16, 26]. In agreement with this mechanism and also proposed to be a contributing factor to the development of cardiomyopathy, hyperglycemia upregulates the expression of E3-ubiquitin ligase CHIP resulting in GATA4 protein degradation. In our study, however, low levels of GATA4 were not associated with any significant increases in the expression of CHIP as previously reported, or in the expression of the ligases MuRF-1 or atrogin1, although increased activity of these ligases has been reported in skeletal muscle of db/db mice [27]. The reasons for these inconsistencies are not clear at the present time, but may relate to differences in insulin resistance, extent of obesity, and the duration and severity of diabetes seen in mice. Regardless, a dual role of CHIP has been documented such as interaction with HSP70, a family of proteins that rescue damaged proteins, prevent stress-dependent apoptosis in heart, and improve heart function in failing ischemic hearts [17, 28]. In transgenic mice lacking the CHIP gene, protein expression of HSP70 in cardiac tissue is reduced following ischemia and ischemic injury is enhanced [17]. Our results showing the low protein expression of HSP70 are consistent with the changes occurring in db/db hearts. Hearts were studied at 14 weeks when apoptosis is present, left ventricular function is depressed, and sensitivity to ischemia is increased [22, 24].

This critical role of GATA4 is supported by the recent observations that GATA4-deleted mice lose the ability for cardiac hypertrophy following pressure overload and exercise stimulation, and overexpression of GATA4 induces cardiac hypertrophy [13, 15]. Loss of GATA4 expression in the adult mouse heart results in a reduction in left ventricular function [13, 29] and activates proapoptotic factors [15]. The down-regulation of cardiac GATA4 protein is consistent with earlier work [16] and we further extend this observation in the db/db heart by demonstrating reduced expression of ANP, BNP, and *α*-MHC. The direct mechanisms leading to reduced expression of these genes are unclear although the consequences on heart structure and function can be substantiated. A reduction in ANP and BNP can alter cardiac structure through it is lack of antihypertrophic and antifibrotic properties in cardiomyocytes, and by inhibition of DNA and collagen synthesis in cardiac fibroblasts [30]. A deficiency in these peptides can also impair diuresis, natriuresis, vasodilation, and inhibit lipolysis [31, 32]. Indeed, both natriuretic peptides are vasodilators and suppress vasoconstrictors such as the renin-angiotensin-aldosterone and sympathetic nervous systems, vasopressin, and endothelin. The increase in systolic pressure observed in diabetic mice would be consistent with low natriuretic peptide status in these mice. In obese patients, recent reports indicate that plasma ANP and BNP levels are reduced which can explain the increased sodium retention and volume expansion in obese patients, and also contribute to the development of heart failure in obesity [33–35]. The lower levels of BNP in diabetes are of relevance especially given recent evidence that transgenic mice that overexpress BNP are lean and resistant to diet-induced obesity, possibly due to increased lipolysis and fat oxidation in adipose cells as well as an upregulation of mitochondrial biogenesis in muscles [36]. It is likely that the presence of a natriuretic peptide deficit may compound the metabolic changes like intramyocardial accumulation of triglycerides and extracellular deposition of excess collagen followed by activation of several signaling pathways [37, 38]. These events can stimulate the myosin isoform switching as reported in this study.

In humans and rodents, the relative expression of *α*- and *β*-MHC isoforms in the heart is controlled by several factors, including the developmental and hormonal milieu, and physiological and pathological states [25, 39–42]. In the failing mouse heart and in models of pressure-induced hypertrophy, a shift from the normally predominant *α*-MHC toward *β*-MHC, the major isoform in contractile function and marker of hypertrophy, is observed [5, 25, 43]. In our study, hearts from diabetic mice exhibited a ~50% decrease in the expression of *α*-MHC and a ~60% increase in *β*-MHC expression. Our data are consistent with the results from an earlier report examining the role of chronic diabetes on *β*-MHC expression using the chemically induced diabetic rat [44]. However, increased expression of *β*-MHC is generally associated with hypertrophy of the heart, which was not the case in db/db mice. Slightly lower heart weights were observed and the heart-weight-to-body-weight ratio was markedly lower in db/db mice. However, in a recent study, we and others have demonstrated that hearts from 16-week-old db/db were atrophied as a result of increased apoptosis [23, 24]. The decrease in the hypertrophy marker NAB1 would be consistent with the progression of atrophy of the heart in this model. Ubiquitination within the heart can also account for the atrophy in view of recent evidence linking atrophy of tissues in the db/db mouse with insulin resistance and excess glucocorticoid synthesis, conditions known to stimulate atrogin1 and MuRF1 production [27, 45–48]. Interestingly, the hypertension observed in db/db mice is not associated with cardiac hypertrophy, suggesting that the increase in systolic pressure may be due to the secondary effects of a low natriuretic peptide status in the db/db mouse.

In conclusion, we show that the GATA4-related regulatory downstream transcripts ANP, BNP, and *α*-MHC are reduced in hearts from db/db mice. This reduction in GATA4 levels was not associated with any increase in the E3-ubiquitin proteosome ligases. However, cardiac levels of the CHIP cochaperone HSP70 levels were decreased in hearts from db/db mice. Together, our results suggest that the downregulation of the GATA4 and associated proteins could in part explain the development of cardiomyopathy in diabetes.

## Figures and Tables

**Table 1:** PCR primer sequences.

Gene	Sense primer (5′–3′)	Antisense primer (5′–3′)	Accession no.
ANP	CCTGTGTACAGTGCGGTGTC	CCTAGAAGCACTGCCGTCTC	NM_008725
BNP	CTGAAGGTGCTGTCCCAGAT	GTTCTTTTGTGAGGCCTTGG	NM_008726
IRAP	CAAAGACCGAGCCAACCTGATC	GCTAAAGAGGAACAACCAGCC	NM_172827
GATA	CACTATGGGCACAGCAGCTCC	TTGGAGCTGGCCTGCGATGTC	NM_008092
*α*-MHC	CTGCTGGAGAGGTTATTCCTCG	GGAAGAGTGAGCGGCGCATCAAGG	NM_001164171
*β*-MHC	TGCAAAGGCTCCAGGTCTGAGGGC	GCCAACACCAACCTGTCCAAGTTC	NM_080728
CHIP	AGGGCAAGGAGGAAAAGGA	TGGCAATGGCCTCATCATAA	NM_019719
Atrogin1	ACTGGACTTCTCGACTGCCAT	CTCCATCCGATACACCCACAT	AF441120
MURF1	AACACAACCTCTGCCGGAA	AGCCCCAAACACCTTGCA	DQ229108
GLUT4	ACCCTGGGCTCTGTATCCC	CCCTGACCACTGAGTGCAAA	AB008453
NAB1	TGCTGACAAGAAGAGATGAG	TCCTGGTTTCCACAGACTAC	NM_008667
GAPDH	TTCACCACCATGGAGAAGGC	GGCATGGACTGTGGTCATGA	NM_008084

ANP: atrial natriuretic peptide; BNP: brain natriuretic peptide; IRAP: insulin-regulated aminopeptidase; G4: GATA binding protein 4; *α*-MHC: alpha-myosin heavy chain; *α*-MHC: beta-myosin heavy chain; CHIP: carboxy terminus of Hsc70-interacting protein; atrogin1: F-box protein 32; MuRF1: muscle RING finger protein 1; GLUT4: glucose transporter protein 4; NAB1: NGF1A-binding protein; GAPDH: glyceraldehyde-3-phosphate dehydrogenase.
